# Quantification of contractile mechanics in the rat heart from ventricular pressure alone

**DOI:** 10.18632/oncotarget.21815

**Published:** 2017-10-10

**Authors:** Chih-Hsien Wang, Ru-Wen Chang, Chun-Yi Chang, Ming-Shiou Wu, Hsien-Li Kao, Liang-Chuan Lai, Tai-Horng Young, Hsi-Yu Yu, Yih-Sharng Chen, Kuo-Chu Chang

**Affiliations:** ^1^ Department of Surgery, National Taiwan University Hospital, Taipei 100, Taiwan; ^2^ Department of Surgery, National Taiwan University Hospital, Hsinchu Branch, Hsinchu 300, Taiwan; ^3^ Department of Physiology, College of Medicine, National Taiwan University, Taipei 100, Taiwan; ^4^ Department of Emergency Medicine, Taipei Veterans General Hospital, Chu-Tung Branch, Hsinchu 310, Taiwan; ^5^ Department of Internal Medicine, National Taiwan University Hospital, Taipei 100, Taiwan; ^6^ Institute of Biomedical Engineering, College of Medicine and Engineering, National Taiwan University, Taipei 100, Taiwan

**Keywords:** cardiac contractility, pressure-ejected volume curve, end-systolic elastance, left ventricular pressure, triangular aortic flow

## Abstract

To quantitate the contractile mechanics of the heart, the ventricle is considered an elastic chamber with known end-systolic elastance (*E_es_*). *E_es_* can be calculated from a single pressure-ejected volume curve, which requires simultaneous records of left ventricular (LV) pressure and the aortic flow (*Q*^m^). In clinical settings, it is helpful to evaluate patients’ cardiac contractile status by using a minimally invasive approach to physiological signal monitoring, wherever possible, such as by using LV pressure alone. In this study, we evaluated a method for determining *E_es_* on the basis of the measured LV pressure and an assumed aortic flow with a triangular wave shape (*Q*^tri^). *Q*^tri^ was derived using a fourth-order derivative of the LV pressure to approximate its corresponding *Q*^m^. Values of *E_es_*^triQ^ obtained using *Q*^tri^ were compared with those of *E_es_*^mQ^ obtained from the measured *Q*^m^. Healthy rats (NC; *n* = 28) and rats with type 1 diabetes (DM; *n* = 26) and chronic kidney disease (CKD; *n* = 20) were examined. The cardiodynamic conditions in both the DM and CKD groups were characterized by a decline in *E_es_*^mQ^ and *E_es_*^triQ^. A significant regression line for *E_es_* was observed (*P* < 0.0001): *E_es_*^triQ^ = 2.6214 + 1.0209 × *E_es_*^mQ^ (*r*^2^ = 0.9870; *n* = 74). Our finding indicates that the systolic pumping mechanics of the heart can be derived from a single LV pressure recording together with the assumed *Q*^tri^.

## INTRODUCTION

**Figure 1:**
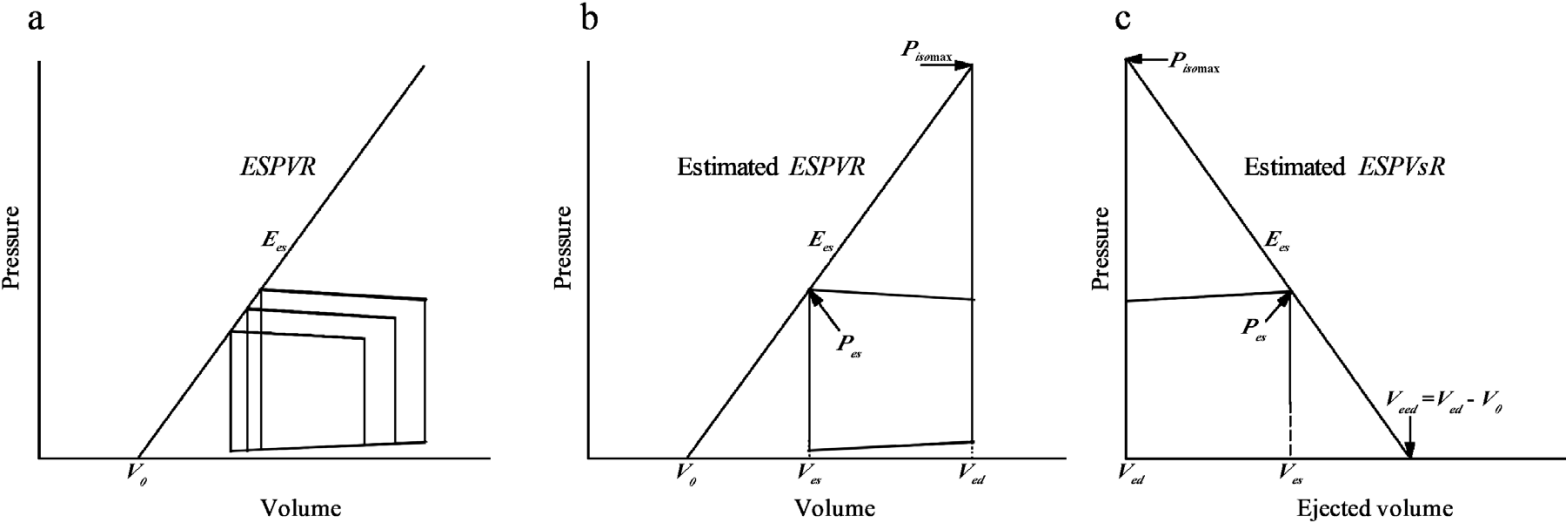
Diagrams to the conventional *ESPVR* determined by a set of three pressure–volume loops **(a),** the estimated *ESPVR* obtained by a single pressure–volume loop on the basis of the estimated *P_iso_*_max_ **(b),** and the estimated *ESPVsR* determined by the pressure–ejected volume curve on the basis of the estimated *P_iso_*_max_ **(c).** *E_es_*, end-systolic elastance; *ESPVR*, end-systolic pressure–volume relationship; *ESPVsR*, end-systolic pressure–stroke volume relationship; *P_es_*, end-systolic pressure; *P_iso_*_max_, peak isovolumic pressure; *V_ed_*, end-diastolic volume; *V_eed_*, effective end-diastolic volume; *V_es_*, end-systolic volume; *V_0_*, zero-pressure volume axis intercept.

The assessment of the cardiac contractile status is important under various physiological and pathological conditions [1]. In the 1960s and 1970s, a simple time-varying elastance model of left ventricular (LV) contraction was proposed to study the intrinsic contractility of the heart, which relates the end-systolic pressure–volume relationship (*ESPVR*) [2–4]. The *ESPVR* of the left ventricle has been reported to be approximately linear over a physiological range, and its slope is the end-systolic elastance (*E_es_*), with the zero-pressure volume axis intercept of *V_0_* [4–6]. Figure 1a illustrates the conventional *ESPVR* line, which is determined by a set of three pressure–volume loops. *E_es_* (but not *V_0_*) markedly varies in response to changes in contractility and is relatively insensitive to changes in preload, afterload, and heart rate (*HR*) in a specific constant contractile status of the heart [4–6].

As noted above, at least three LV pressure–volume loops are required to determine *E_es_* with fairly different end-systolic pressure (*P_es_*) values in a constant inotropic state. Theoretically, if one can estimate the peak isovolumic pressure (*P_iso_*_max_) from an ejecting contraction with reasonable accuracy [7], a single pressure–volume loop would facilitate evaluating the LV end-systolic properties. Figure 1b shows a tangential line connecting the estimated *P_iso_*_max_ to the left corner of the pressure–volume loop, which gives *E_es_* and *V_0_*. Using a nonlinear least-squares approximation technique [7], Takeuchi et al. [8] estimated the isovolumic pressure curve *P_iso_*(*t*) at an end-diastolic volume (*V_ed_*) of an ejecting contraction, successfully evaluating *E_es_* from a single pressure–volume loop in human hearts.

The other method for obtaining *E_es_* entails using the LV end-systolic pressure–stroke volume relationship (*ESPVsR*) from a single pressure–ejected volume curve [9–11], which can be derived from the *ESPVR* [12]. Figure 1c illustrates the LV pressure–ejected volume trajectory for an ejecting beat. The ejected volume curve of the left ventricle can be obtained by the time integration of aortic flow signal. Drawing a tangential line from the estimated *P_iso_*_max_ to the right corner of the pressure–ejected volume curve yields the *ESPVsR*, which gives *E_es_* and its intercept with the ejected volume axis (*V_eed_*). *V_eed_* is the effective LV end-diastolic volume that is the difference between the *V_ed_* and *V_0_*. Thus, the evaluation of ventricular *E_es_* requires simultaneous records of LV pressure and volume to construct a pressure–volume loop or LV pressure and the aortic flow to form a pressure–ejected volume curve.

In clinical settings, it is helpful to evaluate patients’ cardiac contractile status by using a minimally invasive approach to physiological signal monitoring, wherever possible, such as by using LV pressure alone. In the present study, we evaluated a method for determining *E_es_* on the basis of the measured LV pressure and an assumed flow with a triangular wave shape (*Q*^tri^). The unknown *Q*^tri^ was derived using a fourth-order derivative of the LV pressure to approximate its corresponding flow signal measured in the ascending aorta (*Q*^m^). The pressure–ejected volume curve was plotted from the measured LV pressure and time integration of the aortic flow by using either the assumed *Q*^tri^ or measured *Q*^m^. Values of *E_es_*^triQ^ obtained using *Q*^tri^ were compared with those of *E_es_*^mQ^ obtained from *Q*^m^. Healthy rats (NC; *n* = 28) and rats with type 1 diabetes (DM; *n* = 26) and chronic kidney disease (CKD; *n* = 20) were examined.

## RESULTS

**Figure 2:**
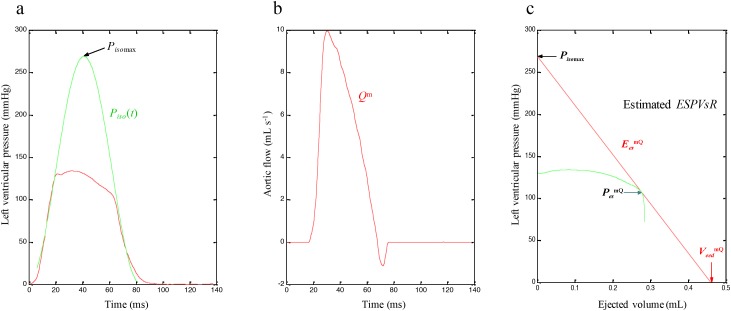
*ESPVsR* line (red line, **c**) estimated from the pressure-ejected volume curve (green line, c), which is obtained by the measured LV pressure (red line, **a**) and time integration of the *Q*^m^ **(b)** in a healthy rat. *ESPVsR*, end-systolic pressure-stroke volume relationship; *E_es_*^mQ^, end-systolic elastance calculated from the LV pressure and *Q*^m^; LV, left ventricular; *P_es_*^mQ^, end-systolic pressure calculated from the LV pressure and *Q*^m^; *P_iso_*, estimated isovolumic pressure curve; *P_iso_*_max_, estimated peak isovolumic pressure; *Q*^m^, measured aortic flow; *V_eed_*^mQ^, effective end-diastolic volume calculated from the LV pressure and *Q*^m^.

Schematic representation of the framework for determining the LV *ESPV_s_R* line from a single pressure–ejected volume curve is illustrated in Figure 1c. Figure 2 exemplifies the pressure-ejected volume curve (green line, c), which is obtained by the measured LV pressure (red line, a) and time integration of the *Q*^m^ (b) in a healthy rat. The *P_iso_*(*t*) curve from an ejecting beat is estimated by the equation 1 described in Methods and is shown as green line in Figure 2a. The *P_iso_*_max_ is the peak pressure point of the estimated *P_iso_*(*t*). Drawing a tangential line from the estimated *P_iso_*_max_ to the right corner of the pressure-ejected volume curve constructs the ventricular *ESPVsR* (red line in Figure 2c) that has the slope of *E_es_*^mQ^ and the volume intercept of *V_eed_*^mQ^.

**Figure 3:**
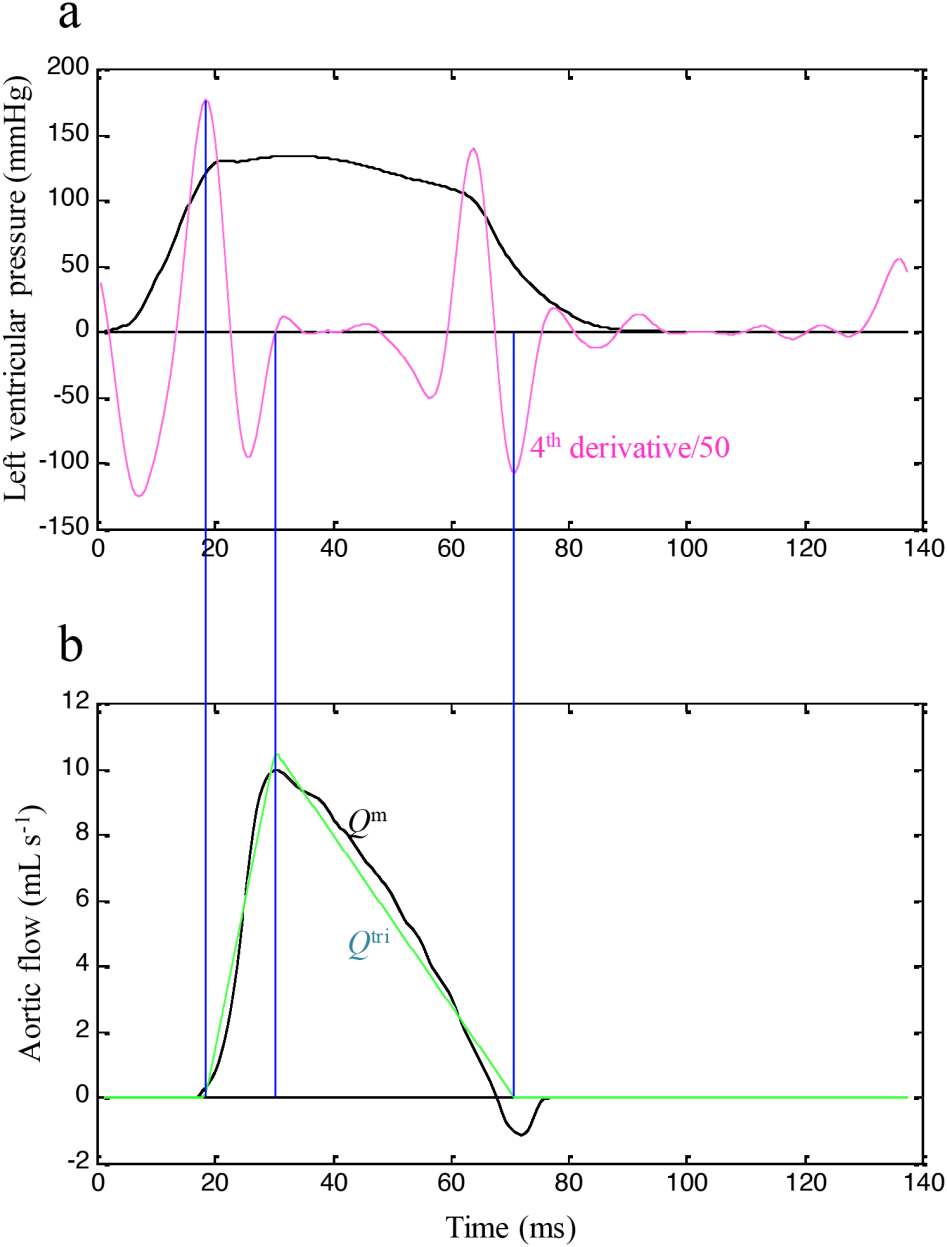
Construction of *Q*^tri^ (green curve, **b**) from the fourth-order derivative (pink curve, **a**) of the measured LV pressure (black curve, a) in the same rat, which is shown in Figure 2. LV, left ventricular; *Q*^m^, measured aortic flow; *Q*^tri^, assumed triangular flow.

Figure 3 exemplifies the construction of *Q*^tri^ (green curve, b) from the fourth-order derivative (pink curve, a) of the measured LV pressure (black curve, a) in the same rat, which is shown in Figure 2. The base of the triangle is constructed with a duration set equal to ejection time. The start and end time points of ejection are identified as the peak of the pink curve near the end of the isovolumic contraction period (first vertical blue line) and the nadir of the pink curve near the middle of the isovolumic relaxation period (third vertical blue line), respectively. After the ejection commenced, the first zero crossing (from negative to positive) determined the peak of the triangle (second vertical blue line). Thus, the *Q*^tri^ is represented by a triangle. After being calibrated using cardiac output (*CO*), the *Q*^tri^ approximates its corresponding *Q*^m^, which is denoted as the black curve in Figure 3b.

**Figure 4:**
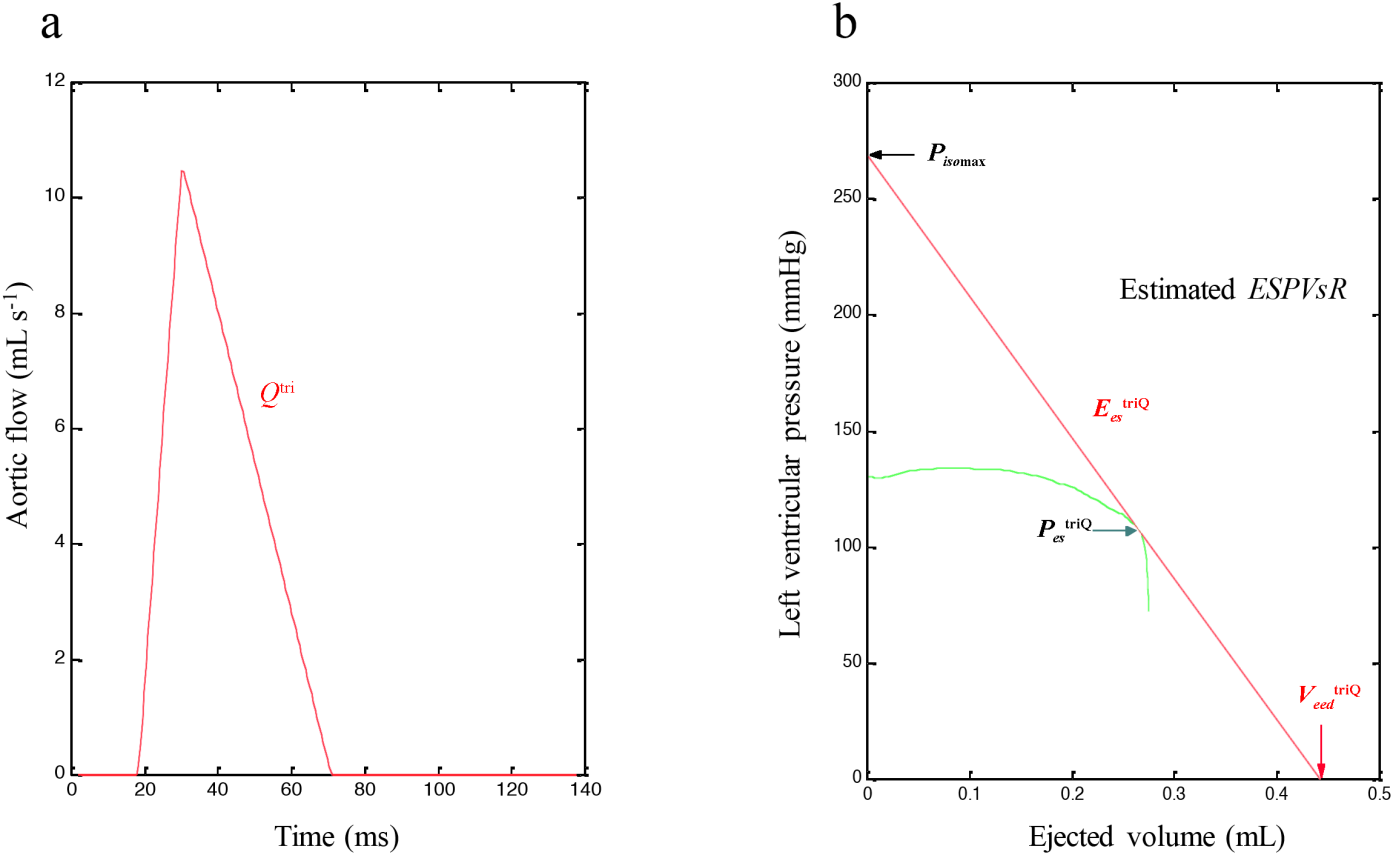
*ESPVsR* line (red line, **b**) estimated from the pressure-ejected volume curve (green line, b), which is obtained by the measured LV pressure (red line in Figure 2a) and time integration of the *Q*^tri^ **(a)** in the same rat, as shown in Figure 2. *ESPVsR*, end-systolic pressure-stroke volume relationship; *E_es_*^triQ^, end-systolic elastance calculated from the LV pressure and *Q*^tri^; LV, left ventricular; *P_es_*^triQ^, end-systolic pressure calculated from the LV pressure and *Q*^tri^; *P_iso_*_max_, estimated peak isovolumic pressure; *Q*^tri^, assumed triangular flow; *V_eed_*^triQ^, effective end-diastolic volume calculated from the LV pressure and *Q*^tri^.

Figure 4 exemplifies the pressure-ejected volume curve (green line, b), which is obtained by the measured LV pressure (red line in Figure 2a) and time integration of the assumed *Q*^tri^ (a) in the same rat, as shown in Figure 2. Figure 2 also shows the LV *P_iso_*_max_ that is generated from the LV pressure by using a nonlinear least-squares approximation technique. Thus, the *ESPVsR* is predicted with the tangential line connecting the estimated *P_iso_*_max_ to the right corner of the pressure–ejected volume curve, which yields *E_es_*^triQ^ and *V_eed_*^triQ^ (Figure 4b).

Measurements were performed in the NC, type 1 DM, and CKD groups. Their baseline characteristics are displayed in Table 1. Compared with the NC group, the type 1 DM group had a higher blood glucose level associated with decreased body weight (BW). Although the DM group showed a decline in *HR* and maximal LV pressure (*P*_max_), no significant change in *CO* and *P_iso_*_max_ was observed in the rats with insulin deficiency. Table 1 also illustrates the impaired renal function in the CKD group, as manifested by the augmented serum creatinine (SCr) and blood urea nitrogen (BUN). Although the CKD group had higher *P*_max_ than the NC group, no alteration in the *HR*, *CO*, and *P_iso_*_max_ was observed in the rats with renal dysfunction.

**Table 1:** Baseline characteristics of healthy rats and rats with type 1 DM and CKD

Group	NC (*n* = 28)	DM type 1 (*n* = 26)	CKD (*n* = 20)
BW (g)	455.0±57.5	320.0±40.0^*^	412.5±60.0
BS (mg dL^-1^)	95.0±10.8	467.5±45.0^*^	na
BUN (mg dL^-1^)	20.3±5.5	na	65.4±14.9^*^
SCr (mg dL^-1^)	0.70±0.10	na	1.70±0.50^*^
*HR* (beats min^-1^)	401.5±50.7	340.1±25.9^*^	386.1±48.7
*CO* (mL s^-1^)	2.277±0.496	2.363±0.564	2.235±0.314
*P*_max_ (mmHg)	138.2±16.0	122.0±16.3^*^	157.1±22.6^*^
*P_iso_*_max_ (mmHg)	269.7±38.2	268.0±46.1	276.3±32.9

All values are expressed as the median ± interquartile range. BW, body weight; BS, blood sugar; BUN, blood urea nitrogen; SCr, serum creatinine; *HR*, basal heart rate; *CO*, cardiac output; *P*_max_, maximal LV pressure; *P_iso_*_max_, peak LV isovolumic pressure; LV, left ventricular; NC, normal controls; type 1 DM, STZ-induced diabetic rats; CKD, rats with chronic kidney disease induced through 5/6 subtotal nephrectomy; na, not applicable.

^*^P < 0.05 compared with controls.

The end-systolic properties of the heart can be described using *P_es_*, *V_eed_*, and *E_es_*. Table 2 shows the effects of DM and CKD on these parameters, as derived from the measured LV pressure and *Q*^m^, namely *P_es_*^mQ^, *V_eed_*^mQ^, and *E_es_*^mQ^. Compared with the NC group, the CKD (but not DM) group exhibited significantly increased *P_es_*^mQ^. Both the DM and CKD groups showed an increase in *V_eed_*^mQ^ and a decrease in *E_es_*^mQ^. Table 2 also illustrates the end-systolic parameters calculated using the measured LV pressure and *Q*^tri^, namely *P_es_*^triQ^, *V_eed_*^triQ^, and *E_es_*^triQ^. Similarly to *P_es_*^mQ^, *P_es_*^triQ^ was augmented by CKD but not DM. The diabetic and CKD groups exhibited deterioration in both *V_eed_*^triQ^ and *E_es_*^triQ^, showing statistical significance similar to that of their measured counterparts (*V_eed_*^mQ^ and *E_es_*^mQ^, respectively).

**Table 2:** Effects of type 1 DM and CKD on *P_es_*^mQ^, *V_eed_*^mQ^, and *E_es_*^mQ^, as derived from the measured LV pressure and *Q*^m^, and their effects on *P_es_*^triQ^, *V_eed_*^triQ^, and *E_es_*^triQ^, as calculated from the measured LV pressure and *Q*^tri^

Group	NC (*n* = 28)	DM type 1 (*n* = 26)	CKD (*n* = 20)
*P_es_*^mQ^ (mmHg)	112.9±11.2	106.2±14.6	144.2±16.4^*^
*V_eed_*^mQ^ (mL)	0.556±0.067	0.728±0.116^*^	0.738±0.179^*^
*E_es_*^mQ^ (mmHg mL^-1^)	483.3±53.0	357.9±74.0^*^	389.5±99.7^*^
*P_es_*^triQ^ (mmHg)	113.9±12.2	106.5±17.4	144.9±15.9^*^
*V_eed_*^triQ^ (mL)	0.544±0.071	0.712±0.125^*^	0.718±0.159^*^
*E_es_*^triQ^ (mmHg mL^-1^)	495.0±50.9	363.0±72.3^*^	395.1±115.4^*^

All values are expressed as the median ± interquartile range. LV, left ventricular; *P_es_*, end-systolic pressure; *Q*^m^, aortic flow measured in the ascending aorta; *Q*^tri^, aortic flow with triangular wave shape; *V_eed_*, effective end-diastolic volume; *E_es_*, end-systolic elastance; NC, normal controls; type 1 DM, STZ-induced diabetic rats; CKD, rats with chronic kidney disease induced through 5/6 subtotal nephrectomy.

^*^P < 0.05 compared with controls.

**Figure 5:**
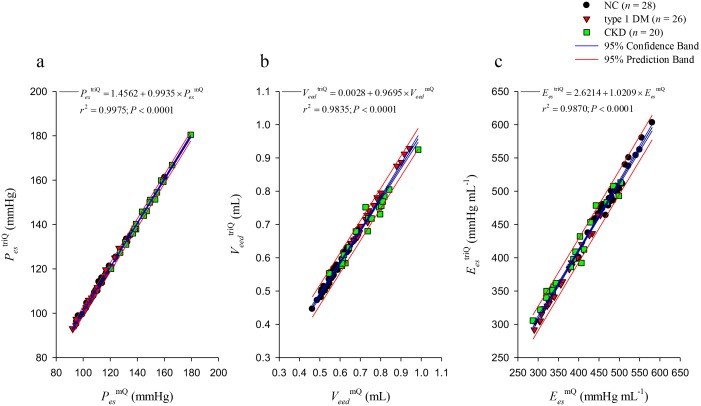
Relations between *P_es_* **(a),** *V_eed_* **(b),** and *E_es_* **(c)** calculated from the measured LV pressure and *Q*^m^ (*P_es_*^mQ^, *V_eed_*^mQ^, and *E_es_*^mQ^, respectively, on the horizontal axes) and *P_es_*, *V_eed_*, and *E_es_* calculated from the measured LV pressure and *Q*^tri^ (*P_es_*^triQ^, *V_eed_*^triQ^, and *E_es_*^triQ^, respectively, on the vertical axes). *E_es_*, end-systolic elastance; LV, left ventricular; *P_es_*, end-systolic pressure; *Q*^m^, measured aortic flow; *Q*^tri^, assumed triangular flow; *V_eed_*, effective end-diastolic volume; NC, normal controls; type 1 DM, streptozotocin-induced diabetic rats; CKD, rats with chronic kidney disease.

Figure 5 displays the relationship of *P_es_*, *V_eed_*, and *E_es_* calculated from the measured LV pressure and *Q*^m^ (*P_es_*^mQ^, *V_eed_*^mQ^, and *E_es_*^mQ^, respectively, on the horizontal axes) with *P_es_*, *V_eed_*, and *E_es_* calculated from the measured LV pressure and *Q*^tri^ (*P_es_*^triQ^, *V_eed_*^triQ^, and *E_es_*^triQ^, respectively, on the vertical axes). Figure 5a shows a significant regression line for *P_es_*: *P_es_*^triQ^ = 1.4562 + 0.9935 × *P_es_*^mQ^ (*r*^2^ = 0.9975; *P* < 0.0001). Figure 5b presents the regression equation of *V_eed_*^triQ^ = 0.0028 + 0.9695 × *V_eed_*^mQ^ (*r*^2^ = 0.9835; *P* < 0.0001). Figure 5c displays the regression line between *E_es_*^triQ^ and *E_es_*^mQ^: *E_es_*^triQ^ = 2.6214 + 1.0209 × *E_es_*^mQ^ (*r*^2^ = 0.9870; *P* < 0.0001). The intercepts of the relationships have no contribution, implying that the relationships are proportional.

**Figure 6:**
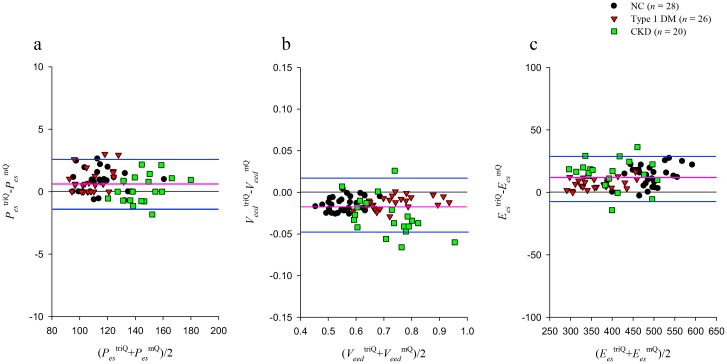
Bland–Altman plots of *P_es_* **(a),** *V_eed_* **(b),** and *E_es_* **(c).** Pink lines represent averages; blue lines denote 95% confidence intervals. *E_es_*, end-systolic elastance; *P_es_*, end-systolic pressure; *V_eed_*, effective end-diastolic volume; NC, normal controls; type 1 DM, streptozotocin-induced diabetic rats; CKD, rats with chronic kidney disease.

Because measurements of the LV end-systolic properties are made using *Q*^tri^ and *Q*^m^ on the same rats, Bland–Altman plots are used to depict the difference between measurements by the two methods. Figure 6 displays the Bland–Altman plots for *P_es_* (a), *V_eed_* (b), and *E_es_* (c), with mean differences of 0.6686 (mmHg), −0.0177 (mL), and 11.3 (mmHg mL^−1^), respectively. The Bland–Altman plots also indicated agreement between the two methods with 95~97% of differences falling between the 95% confidence intervals.

## DISCUSSION

The systolic function of the heart is to supply the appropriate blood flow to the body with its metabolic needs. In patients experiencing cardiac catheterization, cardiac output acting as the systolic function can be measured by the use of the Fick oxygen method [13]. Cardiac output is dependent on the heart rate, myocardial contractility, preload, and afterload [14, 15]. Thus, the systolic function and contractility are not interchangeable [16]. Several investigators have found that the *ESPVR* provides useful information on the contractile status of the heart in evaluating the cardiomyopathy [17, 18]. The pressure-volume ratio can also be used to describe the matching condition for the left ventricle coupled to its arterial system [19]. Thus, measuring the cardiac contractility using the *ESPVsR* could offer an additional value in determining the optimality of energy transmission from the left ventricle to the vasculature [11, 19].

The method we have described provides an estimate of the *E_es_* from a single pressure–ejected volume curve on the basis of the measurement of LV pressure pulse. The major advantages of this method are that it eliminates the requirement for measuring the aortic flow and LV isovolumic contraction. All the calculations are based on the LV pressure waveform obtained from an ejecting beat.

For this study, two steps are demonstrated to generate the ventricular *ESPV_s_R* on the basis of the LV pressure waveform analysis. The first one, based on the fourth-order derivative of the LV pressure, constructed the unknown *Q*^tri^. In 2006, Westerhof et al. [20] described and validated that the aortic flow can be approximated by a triangle. In their study, the timing of the peak triangle was derived using a fourth-order derivative of the aortic pressure waveform [20, 21]. In the present study, the LV pressure was the only signal measured for *E_es_* determination. Based on the fourth-order derivative of the LV pressure, we discovered that the first zero crossing from negative to positive during ventricular ejection can also identify the timing of the peak triangle, which was close to the peak of its corresponding *Q*^m^. After flow calibration, the flow pattern of the assumed *Q*^tri^ was close to that of the measured *Q*^m^ (Figure 3b). Thus, the pressure–ejected volume curve could be obtained from the measured LV pressure and time integration of the assumed *Q*^tri^.

The second step estimated the LV *P_iso_*(*t*), which is another key signal for determining the *ESPV_s_R* from a single pressure–ejected volume curve. The indispensable *P_iso_*(*t*) must be obtained by occluding the ascending aorta at the end of diastole; however, this measuring technique is not permitted in humans. Instead, the method originally described by Sunagawa et al. [7] was used in this study to obtain *P_iso_*(*t*) from the instantaneous pressure of an ejecting contraction. A major concern regarding the estimated *P_iso_*(*t*) is that the duration of the isovolumic contraction caused by abruptly clamping the aortic root is significantly longer than that of the ejecting contraction [22]. Therefore, the cardiac cycle length of the estimated *P_iso_*(*t*) is shorter than that of the measured *P_iso_*(*t*). Despite this observation, Sunagawa et al. [7] reported that the estimated *P_iso_*_max_ is quite close to the *P_iso_*_max_ actually measured by occluding the ascending aorta during diastole. Regarding the estimated *P_iso_*_max_, we discovered that the LV *E_es_* can be approximately calculated using the assumed *Q*^tri^ and that it had a strong correlation with that derived from *Q*^m^ (Figure 5c).

The LV *E_es_* is determined by the ratio of *P_iso_*_max_ to *V_eed_*. Because we observed nonsignificant alterations in *P_iso_*_max_ (Table 1), the increased *V_eed_*^mQ^ was considered the predominant factor for the reduced *E_es_*^mQ^ in the DM and CKD groups (Table 2). A decline in *E_es_*^triQ^ was also reflected by the increase in *V_eed_*^triQ^ in the diabetic and CKD groups (Table 2). Changes in *E_es_*, assessed using either *Q*^m^ or *Q*^tri^, suggested that CKD and DM modify the ventricular chamber properties, thus reducing the inotropic state of the heart. These findings indicate that the method proposed here allows quantification of the contractile mechanics of the heart from the measured LV pressure, in association with an assumed *Q*^tri^, in various diseased animals.

This study had several limitations. Because *E_es_* cannot be measured in conscious animals, evaluating the effects of pentobarbital anesthesia in rats is impossible. The present findings pertain only to the measurements made in anesthetized rats under an open-chest condition. This condition might have induced changes in the LV pressure pulse and introduced reflex effects not found under ordinary conditions. The extent to which anesthesia and thoracotomy influence the cardiac dynamics in rats is uncertain. However, studies conducted on other animals have suggested that the effects on the biological and experimental variability among animals are relatively minor [23].

## MATERIALS AND METHODS

### Animals and catheterization

Two-month-old male Wistar rats were randomly divided into three groups: (1) NC (*n* = 28), (2) type 1 DM (*n* = 26), and (3) CKD (*n* = 20) groups. Type 1 DM was induced using a single tail vein injection with 55 mg kg^−1^ of STZ (Sigma, St. Louis, MO, USA) in 0.1 M citrate buffer (pH 4.5; Sigma) [25]. The blood glucose level was determined using a SURESTEP Test Strip (Lifescan Inc., Milpitas, CA, USA) to confirm hyperglycemia. Under anesthesia with sodium pentobarbital (50 mg kg^−1^; i.p.), CKD was induced through 5/6 subtotal nephrectomy (i.e., right nephrectomy and ligation of two branches of the left renal artery), according to the method reported by Floege et al. [26]. The SCr and BUN levels were measured with an autoanalyzer (Model 7070, Hitachi Electronics Co., Ltd., Tokyo, Japan). Changes in LV end-systolic properties were monitored 8 weeks after DM and CKD induction. All rats were provided ad libitum Purina chow and water and housed under 12-hour light:dark cycles. The experiments were conducted according to the *Guide for the Care and Use of Laboratory Animals*, and our study protocol was approved by the Animal Care and Use Committee of National Taiwan University [25].

Previously described general surgical procedures and methods were used to measure the cardiovascular variables in the anesthetized rats [25]. Briefly, the rats were anesthetized with sodium pentobarbital (50 mg kg^−1^; i.p.), placed on a heating pad, intubated, and ventilated with a rodent respirator (Model 131, New England Medical Instruments, Medway, MA, USA). The chest was opened through the second intercostal space on the right side. An electromagnetic flow probe (100 series; internal circumference, 8 mm; Carolina Medical Electronics, King, NC, USA) was positioned around the ascending aorta to measure *Q*^m^. A high-fidelity pressure catheter (Model SPC 320; size, 2F; Millar Instruments, Houston, TX, USA) was inserted via the isolated right carotid artery into the left ventricle to measure LV pressure. The electrocardiogram (ECG) of lead II was recorded using a Gould ECG/Biotach amplifier (Cleveland, OH, USA). Selective LV pressure and flow signals from 5-10 beats were averaged in the time domain by using the peak R-wave of the ECG as a fiducial point. A single-beat estimation technique was used to evaluate *E_es_* without altering LV loads [8, 10].

### Estimation of the isovolumic pressure from an ejecting contraction

To estimate *P_iso_*(*t*) from an ejecting beat, a nonlinear least-squares approximation technique is used [7]:

$$P_{iso}(t)=\frac{1}{2}P_{id\max}[1-\cos(\omega t\;+\;c)]+P_{d}$$ [1]

*P_id_*_max_ represents the estimated peak isovolumic developed pressure, ω is the angular frequency, *c* is the phase shift angle of the sinusoidal curve, and *P_d_* is the LV end-diastolic pressure. *P_iso_*(*t*) is obtained by fitting the measured LV pressure curve segments from the end-diastolic pressure point to the peak positive *dP_LV_*/*dt* and from the pressure point of the peak negative *dP_LV_*/*dt* to the level same as the end-diastolic pressure of the preceding beat [8]. The peak R-wave of the ECG is used to identify the LV end-diastolic point. Figure 2a schematically represents the relationship between the ejecting contraction (red curve) and estimated isovolumic contraction (green curve) in the pressure–time diagram. *P_iso_*_max_, the estimated peak isovolumic pressure, is the sum of *P_id_*_max_ and *P_d_*.

### Construction of the unknown flow wave with a triangular shape

The unknown *Q*^tri^ was derived from the fourth-order derivative of LV pressure, which is indicated by the pink curve in Figure 3a. *Q*^tri^ onset was identified as the peak of the pink curve near the end of the isovolumic contraction period (first vertical blue line). *Q*^tri^ termination was identified as the nadir of the pink curve near the middle of the isovolumic relaxation period (third vertical blue line). The base of the unknown *Q*^tri^ was subsequently constructed with the duration being same as the time interval between the onset and termination of *Q*^tri^. After the ejection commenced, the first zero crossing from negative to positive (second vertical blue line) determined the peak triangle. The *Q*^tri^ scale was calibrated using the cardiac output. Thus, the unknown flow wave was approximated by a triangular shape (green curve, Figure 3b).

### End-systolic pressure–stroke volume relationship

The LV *E_es_* can be calculated from the *ESPV_s_R* [10, 27]. Briefly, the *P_iso_*_max_ of the left ventricle at the end-diastolic volume is estimated by equation 1. The pressure–ejected volume curve (green line, Figure 2c and 4b) is obtained from the measured LV pressure (red line, Figure 2a) and the time integration of the aortic flow by using either *Q*^m^ (Figure 2b) or *Q*^tri^ (Figure 4a). As shown in Figure 2c and 4b, a tangential line from the estimated *P_iso_*_max_ to the right corner of the pressure–ejected volume curve yields the end-systolic equilibrium point [9, 11]. The line connecting the estimated *P_iso_*_max_ to the aforementioned point is the LV *ESPV_s_R*, which is denoted as the red line. The slope of this line represents the LV *E_es_*, and its intercept with the ejected volume axis is the LV *V_eed_*.

### Statistical analysis

The results are expressed as the median ± interquartile range. For comparing the effect of DM on blood sugar or the effect of CKD on BUN and SCr with that of NC, the Mann-Whitney rank sum test was used to test for a difference between the two groups. However, the Kruskal-Wallis one way analysis of variance (ANOVA) on ranks was performed to determine the statistical significance of the results for the three-group comparison on the LV end-systolic properties. Statistical significance was assumed at the level of *P* < 0.05. In cases where the ANOVA results indicated that a cardiodynamic variable differed significantly among groups, Dunn's test was used to identify which group exhibited divergent median value from that of the NC group.

The simple linear regression uses the equation for a straight line: *y* = *b_0_* + *b_1_x*. *y* is the dependent variable, *x* is the independent variable, *b_0_* is the intercept (or constant term), and *b_1_* is the slope (or regression coefficient). The linearity of the relationship is reflected in the coefficient of determination (*r^2^*). Larger *r*^2^ value (nearer to 1) indicates that the equation is a good description of the relation between the independent and dependent variables. The smaller *P* value denotes the greater probability that the independent variable can be used to predict the dependent variable.

Bland-Altman plots depict the difference between two methods of measurement on the same subjects, in which good agreement is shown by values that lie close to the 0 mean difference line and between the 95% confidence interval limits of agreement [28]. The 95% limits of agreement are estimated by mean difference ± 1.96 standard deviation of the difference.

## CONCLUSIONS

We propose a method for determining the LV *E_es_* from a single pressure–ejected volume curve on the basis of the measured LV pressure and an assumed *Q*^tri^. *Q*^tri^ was derived using a fourth-order derivative of the LV pressure to approximate its corresponding *Q*^m^. The LV *P_iso_*_max_ was generated from an ejecting beat by using a nonlinear least-squares approximation technique. The estimated *P_iso_*_max_ revealed that the LV *E_es_* could be approximately calculated using the assumed *Q*^tri^ and that it had a strong correlation with that derived from *Q*^m^. Our finding indicates that the systolic pumping mechanics of the heart can be derived from a single LV pressure recording together with the assumed *Q*^tri^.

### Perspectives

Our contribution in this endeavor is to provide a path to consider the clinical application of the method estimating the cardiac contractile mechanics from the measured LV pressure alone. The practical advantage of such an approach is that both the *Q*^tri^ and *P_iso_*_max_ are derived from the LV pressure waveform, which is obtained over a single cardiac cycle without any perturbations of the loading conditions. In clinical settings, the *Q*^tri^ scale can be calibrated using the cardiac output, which is easier to measure by noninvasive impedance cardiography, which can be applied quickly, and does not pose a risk of infection, blood loss or other complications [24]. In patients experiencing cardiac catheterization, it is feasible to evaluate the contractile function of the left ventricle by using a minimally invasive measurement on the LV pressure waveform, because the construction of the assumed *Q*^tri^, the generation of the *P_iso_*_max_ and *ESPVsR* line, and the calculation of the LV *E_es_* can be automated.

## Abbreviations

*CO*: cardiac output (mL s^−1^)
*E_es_*: end-systolic elastance (mmHg mL^-1^)
*E_es_*^mQ^: *E_es_* calculated from the LV pressure and *Q*^m^
*E_es_*^triQ^: *E_es_* calculated from the LV pressure and *Q*^tri^
*ESPVR*: end-systolic pressure-volume relationship
*ESPV_s_R*: end-systolic pressure-stroke volume relationship
*HR*: basal heart rate (beats min^−1^)
LV: left ventricular
*P_es_*: end-systolic pressure (mmHg)
*P_es_*^mQ^: *P_es_* calculated from the LV pressure and *Q*^m^
*P_es_*^triQ^: *P_es_* calculated from the LV pressure and *Q*^tri^
*P_iso_*: isovolumic pressure curve (mmHg)
*P_iso_*_max_: peak isovolumic pressure (mmHg)
*P*_max_: maximal pressure (mmHg)
*Q*^m^: measured aortic flow wave (mL s^−1^)
*Q*^tri^: calibrated triangular flow wave (mL s^−1^)
*V_ed_*: end-diastolic volume (mL)
*V_eed_*: effective end-diastolic volume (mL)
*V_eed_*^mQ^: *V_eed_* calculated from the LV pressure and *Q*^m^
*V_eed_*^triQ^: *V_eed_* calculated from the LV pressure and *Q*^tri^
*V_0_*: zero-pressure volume axis intercept (mL)
